# Tracking the Trends and Projection of Pediatric Malnutrition Towards Global Nutrition Targets by 2030—A Secondary Data Analysis of Low Middle-Income Countries

**DOI:** 10.3390/nu18071160

**Published:** 2026-04-04

**Authors:** Asif Khaliq, Bushra Ashar, Amreen, Safi Ullah Khan, Muhammad Junaid, Angus Ruggieri-Guthrie, Mohammad Javad Davoudabadi, Shafaq Taseen, Maryam Ranta, Mezhgan Kiwan, Nazeer Ahmed, Haji Abdul Rehman Akhter

**Affiliations:** 1 School of Public Health and Social Work, Queensland University of Technology, Brisbane 4059, Australia; 2 Waikato District Health Board, Health New Zealand–Te Whatu Ora, Hamilton 3204, New Zealand; 3 Department of Surgery, Dow University of Health Sciences (DUHS), Karachi 74200, Pakistan; bushra_qaiser84@hotmail.com; 4 Department of Psychology, University of Karachi, Karachi 75270, Pakistan; amreen@uok.edu.pk; 5 Medical and Dental College, Bahria University, Karachi 75260, Pakistan; rsafiullah3@gmail.com; 6 Department of Medicine, Lady Reading Hospital, Peshawar 25000, Pakistan; junaidkhanx55@gmail.com; 7 School of Public Health, University of Queensland, Brisbane 4072, Australia; ruggieriguthrie@gmail.com; 8 School of Mathematical Sciences, Queensland University of Technology, Brisbane 4000, Australia; mohammad.davoudabadi@qut.edu.au; 9 Department of Paediatrics and Child Health, Division of Woman and Child Health, Aga Khan University, Karachi 74800, Pakistan; s.angilahasan@gmail.com; 10 Department of Medicine, Karachi Medical and Dental College, Karachi 74700, Pakistan; maryamranta@gmail.com (M.R.); nazeermed313@gmail.com (N.A.); 11 Department of Medicine, Gomal Medical College, Dera Ismail Khan 29050, Pakistan; kiwanmezhgan@gmail.com; 12 Department of Medicines, CMH Multan Institute of Medical Sciences, Multan 60000, Pakistan; abdulrehman.med@gmail.com

**Keywords:** malnutrition, stunting, wasting, obesity, children, coexisting forms, trends, global nutrition targets (GNTs), low- and middle-income countries (LMICs), Sustainable Development Goals (SDGs)

## Abstract

**Objective:** This study aimed to estimate the trends, projections, and determinants of standalone and *coexisting forms of malnutrition* (CFM) at the global, regional, national, and individual level among children under five in *low- and middle-income countries* (LMICs). It also assessed the projection trajectory towards the 2030 *global nutrition targets (GNTs)* for child growth including stunting, wasting, obesity, and CFM. **Methods:** Data from 48 LMICs were analyzed using the Multiple Indicator Cluster Surveys (MICS) and Demographic and Health Surveys (DHS). Children with complete anthropometry were included for national- and individual-level descriptive analyses. Projected prevalence of each form of malnutrition, including CFM, was calculated using the Annual Rate of Change. Inferential analyses employed generalized linear regression models with two-way interaction terms to identify determinants of each malnutrition type. **Findings:** By 2030, 22 of 48 LMICs are projected to achieve the GNT of stunting, wasting, and obesity, that is up from 10 countries currently, while Yemen and Zimbabwe are expected to remain off-track. Stunting is the most prevalent form, affecting 42 countries, with nine nations projected to have over 50% of children affected by a form of malnutrition. Wasting, obesity, and CFM are rising in several countries. Maternal education and household wealth were the strongest determinants, with children of uneducated mothers and from poorest households at the highest risk. Inequalities are narrowing slowly by 1–2% per year, and marked regional disparities persist. **Conclusions:** Many LMICs are off-track to meet child-growth targets when CFM is considered alongside standalone indicators. The government and global health partners must strengthen nutrition surveillance systems and equity-focused policies and programs to routinely capture CFM and prevent as well as manage all forms of malnutrition at the national and individual levels.

## 1. Introduction

Malnutrition is one of the pressing public health concerns contributing substantially to around half of the deaths among children under five years of age [1]. It encompasses a variety of conditions, such as undernutrition, overnutrition and micronutrient disturbance (i.e., deficiency or excess) [2]. The recent *Joint Malnutrition Estimates* (JME) report of 2024 proposed by the UNICEF, WHO and World Bank underscored 23.2% stunting, 6.6% wasting and 5.5% overweight cases for children aged below five years [3]. This contributes to global malnutrition prevalence in more than a third of children below age five years.

The global burden of malnutrition among children under five years of age is not homogenous. Children living in *low- and middle-income countries* (LMICs) have disproportionally high prevalence of malnutrition [3]. In general, children living in Asia and Africa are highly vulnerable to various forms of malnutrition, where more than half of children are susceptible to different types of nutritional adversities [4]. Poverty, food insecurity, limited access to quality healthcare, and inadequate water and sanitation systems continue to perpetuate the cycle of undernutrition and emerging overnutrition in these countries [3].

Malnutrition in children has profound consequences on their health and development. Pediatric malnutrition has a synergistic relationship with children’s illness, including disability and cognitive impairment, which in turn are associated with negative economic outcomes [5]. Owing to this reason, controlling and eliminating malnutrition, specifically among young children, has been recognized as an urgent public health concern by various local and international bodies [3,6]. To combat malnutrition, several nutrition-specific and nutrition-sensitive interventions were employed by different countries [7,8]. Furthermore, the Millennium Development Goals (MDGs) and Sustainable Development Goals (SDGs) have set various indicators and targets for eliminating malnutrition by the year 2030 [9,10]. Similarly, the *global nutrition targets* (GNTs) also have set targets for reducing stunting to 40%, keeping wasting below 5%, and halting the rise in pediatric obesity by 2030 [11]. However, the progress of most countries for reducing malnutrition is not meeting the global malnutrition targets, and malnutrition is highly prevalent among the children of LMICs. Approximately 59% of global stunting cases, 73% of global wasting cases, and 35% of global overweight cases of children under five years reside in LMICs [3]. That depicts either slow or no progress to combat pediatric malnutrition in LMICs by 2030. There is a pressing need for robust forecasting models that can better evaluate progress toward achieving the WHO-GNT and SDG-2.2 for each country [11,12,13].

The nutrition related indicators, targets and goals devised globally are focused on tracking the progress of standalone forms of malnutrition at the national level. However, the prevalence of *coexisting forms of malnutrition* (CFM) and its various types in an individual are not yet assessed by any local and international bodies. Therefore, this study tracked the trends and projected the prevalence and determinants of malnutrition and its different types, including CFM, among the neonates, infants and young children across 48 LMICs, using combined Demographic and Health Surveys (DHS) and Multiple Indicator Cluster Surveys (MICS) by employing a generalized linear regression model (GzLM).

## 2. Methodology

### 2.1. Study Design and Data Source

In this study, nationally representative datasets retrieved from the DHS and MICS databases were utilized for tracking and forecasting the malnutrition burden among children under five years of age. The DHS program was first promulgated in 1984 by the United States Agency for International Development (USAID), while the MICS survey program was started in 1995 by the UNICEF [14,15]. Both the DHS and MICS collected data related to demography, basic health, nutrition, and development indicators from over 90 LMICs [16]. These datasets provide evidence to the program manager, policy makers and other stakeholders about the global development progress of different health and nutrition-related indicators including those proposed in MDGs, Universal Health Coverage (UHC), and SDGs.

### 2.2. Study Population and Eligibility Criteria

This study targeted the anthropometric data of children aged between 0 and 59 months belonging to LMICs. A multistep inclusion and exclusion criteria were applied in this study. Firstly, a list of all countries of the world irrespective of their income classification was created. The list contained six different regions as per the World Health Organization (WHO) classification; the African Region (AFRO) with 47 countries, the Region of the Americas (AMRO/PAHO) with 35 countries, the South-East Asia Region (SEARO) with 11 countries, the European Region (EURO) with 53 countries, the Eastern Mediterranean Region (EMRO) with 22 countries, and the Western Pacific Region (WPRO) with 27 countries [17]. From this WHO regional classification list, LMICs were selected, as per the World Bank Income classification FY-2025 [18]. In the second step, data for all the LMICs and LICs were retrieved either from the DHS or MICS database. For inclusion, LMICs and LICs must have at least two datasets in total, with one around the year 2012 (±5 years) and the other any time after 2012. In addition, data from each country must have anthropometric variables reported to be included in the study. All upper-income countries (UICs) and countries which had either one dataset or incomplete anthropometry were excluded. Furthermore, data of certain countries that were classified as an LMIC at time of data collection but are now removed from LICs and LMICs list were also excluded.

### 2.3. Conceptual Framework

**Figure 1:**
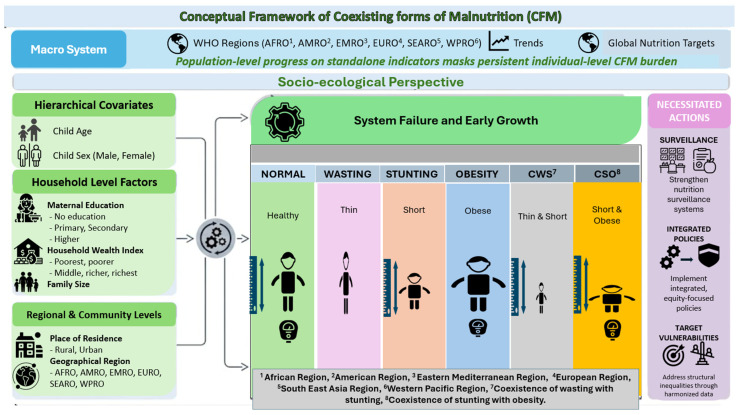
Conceptual framework of coexisting forms of malnutrition.

The analysis of this study adopted a socio-ecological approach to child malnutrition, recognizing that CFM arises from interactions between the immediate- (age, sex), household- (maternal education, wealth, family size), community- (urban/rural residence), and macro-level (WHO region, temporal trends) determinants acting across the first 1000 days of life, including feeding indicators [19]. CFM is conceptualized as a marker of nutritional transition and systems failure. Chronic stunting from early linear growth faltering coincides with acute wasting due to shifting dietary quality and care practices within the same population. The framework links observed patterns to GNT, thereby hypothesizing that population-level progress on standalone indicators masks persistent individual-level CFM burden. This necessitates surveillance, interventions, and structural vulnerabilities that are captured through harmonized covariates (Figure 1).

### 2.4. Study Outcomes

In this study, tracking and forecasting of the nutritional profiles of young children below five years was carried out by measuring their anthropometric variables such as stunting, wasting and obesity. According to the WHO, a child is considered as stunted and wasted, if the z-score value for height-for-age (HAZ) and weight-for-*height* (WHZ) is ≤−2.00 S.D., respectively [20]. However, children with a WHZ value ≥ 2.00 S.D. are considered as obese [21]. Children experiencing more than one type of nutritional disorder would be considered to have CFM [22,23]. The *Global Nutritional Report* (GNR) proposed two major types of CFM in children aged below five years, such as *coexistence of stunting with obesity (CSO)* and *coexistence of wasting with stunting (CWS)* [22].

### 2.5. Study Covariates

A wide range of biological, social, cultural, economic, ecological, and geographical factors may influence the prevalence and trends of malnutrition, including CFM. Based on data availability and completeness within the selected datasets, relevant covariates were identified and organized into a hierarchical framework comprising individual-, household-, and regional/community-level factors.

At the individual level, covariates included the child’s age and sex (male, female). Household-level factors comprised maternal education (no education, primary, secondary, higher) and household wealth index (poorest, poorer, middle, richer, richest). Regional- and community-level factors included place of residence (rural, urban) and geographical region (AFRO, AMRO, EMRO, EURO, SEARO, WPRO).

### 2.6. Data Handling and Management

After data retrieval, data screening was carried out, in which a team of researchers (AK, BA, MR, MK) reviewed all the variables and identified variables of interest: HAZ, WHZ, and basic variables such as age, sex, education, wealth index and region. The datasheet, which contains all the variables of interest, was included, while those devoid of variables of interest were excluded. Following data screening, the standardization of each dataset was performed. The dataset standardization is characterized by the homogenization of each variable and its categories, i.e., heterogeneous categories or differing categories of each variable were converted to homogeneous categories. Some of the variables, such as the education and wealth index, have non-standardized categories, and these heterogeneous categories were transformed into homogeneous categories. For example, in DHS, the wealth index has five categories: poorest, poorer, middle, richer and richest, and MICS data also has five wealth index categories: poorest, second, middle, fourth and richest. The wealth index coding and the coding order were similar in both datasets, ranging from 1 to 5. Regarding the education, the DHS datasets have four categories: no education, primary education, secondary education, and higher education. The MICS datasets showed six education categories: none, pre-primary, primary, secondary, higher and tertiary. The six education categories of the MICS datasets were converted into four categories according to the DHS education categories. However, some of the variables, such as sex and region, have homogenous categories and homogenous coding in all the datasets. Certain variables, such as child age and their z-score values, were converted from a continuous variable to a categorical variable. Additional variables were created in each dataset, of which some were created from the existing variables, i.e., a new variable called ‘nutritional status’ was created from the HAZ and WHZ variables, and this new variable consists of six different types of nutritional status: normal, wasting, stunting, obesity, CWS, and CSO. However, some variables, such as survey year, dataset type (DHS and MICS), and geographical region (AFRO, AMRO, EURO, EMRO, SEARO, and WPRO), were added in each dataset for identification purposes. Finally, data merging was carried out for each country, followed by each region as defined by the WHO and finally the globe.

### 2.7. Statistical Analysis

The analyses of this study were conducted using pooled, nationally representative survey datasets from baseline and current rounds across 48 LMICs. Sampling weights, clustering, and stratification variables were incorporated throughout to ensure nationally representative estimates for the complex survey design.

Initially, data was analyzed descriptively using Statistical Package for the Social Sciences—Version 29 (SPSS-29). The prevalence of each nutrition indicator, stunting, wasting and obesity (Table 1), and various forms of malnutrition such as stunting, wasting, obesity, CWS, and CSO (Table 2) were calculated separately for baseline and current survey rounds. Annual Rate of Change (ARR) was calculated for each nutritional indicator and malnutrition type using Microsoft Excel-365 (Supplementary Files S1 and S2). This assessed the temporal change between two survey rounds and is widely used globally in nutrition-monitoring frameworks. The JME produced by UNICEF, WHO, and the World Bank used this technical approach ensuring methodological comparability with GNTs. ARR was derived using following formula:

ARR = ln(P_2_/P_1_)/t

where

$P_{1}$ = prevalence at baseline survey;

$P_{2}$ = prevalence at current survey;

t = time interval (years) between surveys;

ln = natural logarithm.

Based on the ARR value, the projected prevalence of each nutrition indicator and each form of malnutrition was estimated using following formula:

Projected Prevalence = P_2_ × e(ARR × t)

where

t = time interval (years) from the most recent survey to 2030.

The inferential analyses were performed using a Generalized Linear Model (GzLM) framework with binomial logistic regression. Six mutually exclusive nutritional states were modeled separately: normal nutritional status (reference category), stunting, wasting, obesity, CWS and CSO. The logit link function was specified for categorical outcomes. GzLM was selected due to its flexibility in link functions, robustness for large datasets and suitability for binary and multinomial outcomes. Robust standard errors were estimated to account for clustering and heteroskedasticity. Furthermore, model assumptions of logistic regression were assessed and satisfied for independence of observations, correct model specification, absence of multicollinearity (VIF observed <5), linearity of continuous predictors, and large sample size. The model diagnostics indicated good fitness and no violation of key assumptions.

To examine temporal shifts in disparities, interaction terms between each covariate and survey year (covariate × year) were incorporated. These interactions assessed whether socioeconomic, demographic, and regional gradients across two survey periods changed for each region. Additionally, interaction terms between survey year and malnutrition type were evaluated to determine whether temporal trends differed across nutritional states. The GzLM framework enabled straightforward inclusion of these interaction terms while maintaining computational efficiency and valid inference under complex survey design.

### 2.8. Ethical Considerations

This study utilized published and anonymous data from the MICS and DHS databases. Hence, ethical approval was not required for this study according to the international standards.

## 3. Results

### 3.1. Scenario-Based Projections of GNTs to 2030 Among Children Under Five Across LMICs

Table 1 described the GNTs of each form of pediatric malnutrition (stunting, wasting and obesity) across two survey periods, i.e., baseline and current. Additionally, it presents the ARR of projected prevalence for each nutritional indicator to the year 2030.

By 2030, five (Comoros, Gambia, Malawi, Nigeria, and Sao Tome and Principe) out of 48 LMICs would be expected to meet the GNTs of reducing stunting by 40%, bringing wasting below 5%, and bringing obesity below the existing level. Projections for 2030 indicate that 17 (Bangladesh, Benin, Burkina Faso, Côte d’Ivoire, Ghana, Guinea-Bissau, Honduras, Kenya, Kyrgyzstan, Mali, Mozambique, Nepal, Pakistan, Rwanda, Sierra Leone, Tajikistan, and Togo) countries will be expected to meet two cut-offs for nutritional thresholds. Twenty-three countries (Burundi, Cameron, Chad, Congo, DRC, Egypt, Eswatini, Ethiopia, Guinea, Haiti, Laos, Lesotho, Liberia, Madagascar, Mauritania, Palestine, Senegal, Sudan, Tunisia, Uganda, Timor-Leste, Yemen, and Zambia) will be expected to meet the threshold of one GNT. However, India, Jordan and Zimbabwe are projected not to meet any GNTs. India and Zimbabwe both will be expected to reduce the prevalence of pediatric stunting by 2030, but the progress to reduce stunting in both countries will not be enough to reach the GNT of 40% stunting reduction.

Of the 48 LMICs included, the prevalence of stunting in children will be expected to decrease in 39 countries. Of which, 21 countries (Bangladesh, Burkina Faso, Comoros, Côte d’Ivoire, Egypt, Eswatini, Ethiopia, Gambia, Ghana, Kenya, Laos, Malawi, Mali, Nepal, Nigeria, Pakistan, Rwanda, Sao Tome and Principe, Togo, Tajikistan, and Timor-Leste) will be expected to show 40% reduction in stunting prevalence by 2030. However, the prevalence of stunting will be expected to increase over the baseline stunting level in nine countries: Benin, Cameron, DRC, Guinea-Bissau, Jordan, Liberia, Sudan, Tunisia and Yemen. 

More than half of LMICs (approximately 33 countries) are expected to reduce the wasting prevalence in children from the baseline wasting prevalence by over 5%; however, 19 countries (Benin, Cameron, Comoros, Gambia, Guinea-Bissau, Haiti, Honduras, Kyrgyzstan, Lesotho, Liberia, Malawi, Mozambique, Nigeria, Palestine, Rwanda, Sao Tome and Principe, Sierra Leone, Uganda, Zambia) will be expected to reduce the wasting prevalence to less than 5% in 2030. The baseline and current prevalence of wasting in Eswatini, Jordan and Tunisia is within 5%, but by 2030 it would be expected to increase. Similarly, in Chad, Congo, Egypt, Ethiopia, Kenya, Laos, Mauritania, Senegal, Timor-Leste, Togo, Yemen and Zimbabwe, more than 5% of children will be expected to suffer from wasting by 2030.

Children living in LMICs also experience obesity. Currently, 13 LMICs report obesity prevalence exceeding the global threshold of 3%. Certain LMICs have a high obesity threshold at baseline that now meets the GNT of <3% obesity. These included Cameron, Egypt, Haiti, India, Jordan, Laos, Lesotho, Liberia, Palestine, Rwanda, Timor-Leste, Uganda, and Zambia. By 2030, over 30 LMICs are positioned to achieve the GNT for childhood obesity. Despite this positive trajectory, a subset of 14 countries is expected to maintain an obesity prevalence exceeding the 3% threshold. These findings highlight a persistent public health challenge in these specific regions contrasting with the broader progress observed across other LMICs.

**Table 1:** Scenario-based projections of GNTs to 2030 among children under five across LMICs.

	Survey Period	Sample Size	Stunting		Wasting		Obesity	
			Prevalence	Status	Prevalence	Status	Prevalence	Status
**African Region (AFRO)**								
**Benin**	**2012**	7636	44.6	**↑**	17.6	**↓**	17.6	**↓**
	**2018**	11,631	52.7		7.2		1.9	
	2030 *		62.3		2.9		0.2	
**Burundi**	**2010**	3449	55.7	**↓**	7.7	**↓**	2.9	**↓**
	**2016**	6039	55.2		7.2		1.6	
	2030 *		54.8		6.7		0.9	
**Burkina Faso**	**2014**	6532	35.2	**↓**	16.9	**↓**	2.2	**↓**
	**2021**	5712	22.4		11.5		1.6	
	2030 *		14.3		7.8		1.2	
**Cameron**	**2011**	5033	32.7	**↑**	7.2	**↓**	6.5	**↑**
	**2022**	4445	39.2		5.3		10.8	
	2030 *		47		3.9		17.9	
**Chad**	**2010**	12,280	40.1	**↓**	16.2	**↑**	0	**↓**
	**2019**	7188	31.9		20.1		0	
	2030 *		25.2		24.9		0	
**Comoros**	**2012**	2387	28.6	**↓**	13.4	**↓**	8.8	**↓**
	**2022**	4464	16.9		4.9		0	
	2030 *		10		1.8		0	
**Congo**	**2012**	4475	28	**↓**	7.3	**↑**	3.4	**↓**
	**2015**	4895	25.6		8.1		0	
	2030 *		23.4		9		0	
**Côte d’Ivoire**	**2012**	3200	31	**↓**	9.1	**↓**	3	**↓**
	**2021**	5056	21		8.2		0	
	2030 *		14.2		7.4		0	
**DRC**	**2010**	11,093	41.1	**↑**	8.1	**↓**	4.6	**↓**
	**2018**	21,456	43.2		7		0	
	2030 *		45.4		6		0	
**Eswatini**	**2010**	2647	30.2	**↓**	0.8	**↑**	0	**↓**
	**2022**	2165	20.1		1.8		0	
	2030 *		13.4		4.1		0	
**Ethiopia**	**2011**	9611	43.1	**↓**	13.4	**↑**	1.8	**↑**
	**2016**	8768	37.5		13.8		2.1	
	2030 *		32.6		14.2		2.5	
**Gambia**	**2013**	3098	27.1	**↓**	13.4	**↓**	2.8	**↓**
	**2020**	3805	19.4		7		1.8	
	2030 *		13.9		3.7		1.2	
**Ghana**	**2014**	2379	28.4	**↓**	11.1	**↓**	4.9	**↓**
	**2022**	4395	19.8		7.5		1.8	
	2030 *		13.8		5.1		0.7	
**Guinea**	**2012**	3085	32.2	**↓**	11.9	**↓**	33.6	**↓**
	**2018**	3371	31.9		10.5		5.5	
	2030 *		31.6		9.3		0.9	
**Guinea-Bissau**	**2014**	7573	26.7	**↑**	5.8	**↓**	0	**↓**
	**2019**	7484	26.9		4.8		0	
	2030 *		27.1		4		0	
**Kenya**	**2014**	5096	34.8	**↓**	6.7	**↑**	5.2	**↓**
	**2022**	17,283	19.3		9		2.7	
	2030 *		10.7		12.1		1.4	
**Lesotho**	**2014**	1623	40.4	**↓**	6.1	**↓**	7.3	**↓**
	**2024**	1089	38.3		3.6		6.7	
	2030 *		34		2.1		6.1	
**Liberia**	**2013**	31,717	32	**↑**	8.8	**↓**	2.6	**↑**
	**2020**	2440	33		6.4		4.3	
	2030 *		34		4.7		7.1	
**Madagascar**	**2009**	4861	48.9	**↓**	10.9	**↓**	5.2	**↓**
	**2021**	5756	39.6		9.2		1.9	
	2030 *		32.1		7.8		0.7	
**Malawi**	**2010**	4856	47.1	**↓**	6	**↓**	8	**↓**
	**2016**	5110	36.2		5		4.4	
	2030 *		27.8		4.2		2.4	
**Mali**	**2013**	4306	38.7	**↓**	14.3	**↓**	2.2	**↓**
	**2018**	8223	27.8		11.4		2.2	
	2030 *		20		9.1		2.2	
**Mauritania**	**2011**	9278	27	**↓**	13.4	**↑**	0	**↓**
	**2015**	10,655	25.8		13.8		0	
	2030 *		24.7		14.2		0	
**Mozambique**	**2011**	9313	40.3	**↓**	7	**↓**	7.8	**↓**
	**2023**	37,230	33.9		5.1		3.6	
	2030 *		28.5		3.7		1.7	
**Nigeria**	**2013**	19,010	43.2	**↓**	15.8	**↓**	9.1	**↓**
	**2018**	11,314	37.2		8.7		2	
	2030 *		32		4.8		0.4	
**Rwanda**	**2013**	4075	44.5	**↓**	4.7	**↓**	6.9	**↓**
	**2020**	3805	34.7		3.1		5.7	
	2030 *		27.1		2		4.7	
**Sao Tome and Principe**	**2014**	2030	17	**↓**	4.1	**↓**	0	**↓**
	**2019**	1832	13.1		4.1		0	
	2030 *		10.1		4.1		0	
**Senegal**	**2011**	5882	21.5	**↓**	11.7	**↑**	1.4	**↓**
	**2023**	4466	20.5		12.7		1	
	2030 *		19.5		13.8		0.7	
**Sierra Leone**	**2013**	2024	35.2	**↓**	12.5	**↓**	9.4	↓
	**2019**	4100	31.4		7.3		4.7	
	2030 *		28		4.3		2.4	
**Togo**	**2010**	4625	33.7	**↓**	5.5	**↑**	0	**↓**
	**2017**	4900	24.6		5.8		0	
	2030 *		18		6.1		0	
**Uganda**	**2011**	2070	33.5	**↓**	7.4	**↓**	4.1	**↓**
	**2019**	4390	29.5		5.4		4	
	2030 *		26		3.9		3.9	
**Zambia**	**2013**	11,407	40.4	**↓**	8	**↓**	5.8	**↓**
	**2018**	8681	35.5		6		5.2	
	2030 *		31.1		4.5		4.7	
**Zimbabwe**	**2011**	4299	32.7	**↓**	5.2	**↑**	5.6	**↑**
	**2015**	4914	26.5		5.6		6.3	
	2030 *		21.5		6		7.1	
**American Region (AMRO)**								
**Haiti**	**2012**	3984	23.6	**↓**	6.8	**↓**	3.6	**↓**
	**2017**	5583	22.9		5.6		3.5	
	2030 *		22.2		4.6		3.4	
**Honduras**	**2012**	9973	26.9	**↓**	3.5	**↓**	5	**↓**
	**2019**	8466	19.7		1.8		0	
	2030 *		14.4		0.9		0	
**Eastern Mediterranean Region (EMRO)**								
**Egypt**	**2008**	9477	29.4	**↓**	8.4	**↑**	18.4	**↓**
	**2014**	13,682	21		11.7		12.7	
	2030 *		15		16.3		8.8	
**Jordan**	**2012**	6266	8.8	**↑**	2.3	**↑**	5	**↑**
	**2023**	4927	9.3		2.4		9.1	
	2030 *		9.8		2.5		16.6	
**Pakistan**	**2012**	3071	45.1	**↓**	10.4	**↓**	6.8	**↓**
	**2017**	4098	38.6		8		3.1	
	2030 *		33		6.2		1.4	
**Palestine**	**2010**	7832	11	**↓**	3.2	**↓**	5.4	**↑**
	**2020**	4646	8.7		1.4		9.7	
	2030 *		6.9		0.6		17.4	
**Sudan**	**2010**	11,601	35.8	**↑**	16.1	**↓**	0	**↓**
	**2014**	10,800	39.8		15.4		0	
	2030 *		44.2		14.7		0	
**Tunisia**	**2011**	2563	10.5	**↑**	2.5	**↑**	0	**↓**
	**2023**	1925	12.6		3.1		0	
	2030 *		15.1		3.8		0	
**Yemen**	**2013**	13,624	46.8	**↑**	17.6	**↑**	2.1	**↓**
	**2023**	17,744	48.9		18.5		0	
	2030 *		51.1		19.4		0	
**European Region (EURO)**								
**Kyrgyzstan**	**2014**	4427	13.2	**↓**	2.2	**↓**	0	**↓**
	**2018**	3545	10.8		2		0	
	2030 *		8.9		1.8		0	
**Tajikistan**	**2012**	4523	26.2	**↓**	12	**↓**	5.3	**↓**
	**2017**	5844	19.6		8.2		3.9	
	2030 *		14.7		5.6		2.9	
**South-East Asian Region (SEARO)**								
**Bangladesh**	**2011**	7647	41.5	**↓**	17.1	**↓**	1.6	**↓**
	**2022**	4087	24.5		13.2		1.5	
	2030 *		14.5		10.2		1.4	
**India**	**2015**	225,002	39.4	**↓**	21.8	**↓**	2.3	**↑**
	**2021**	198,802	36.9		20		3.7	
	2030 *		34.7		18.3		6	
**Nepal**	**2011**	2335	42.9	**↓**	12.2	**↓**	1.2	**↓**
	**2022**	2586	28.4		9		1.2	
	2030 *		18.8		6.6		1.2	
**Timor-Leste**	**2010**	7544	57.9	**↓**	20.9	**↑**	4.3	**↑**
	**2016**	5557	47.4		25.5		5	
	2030 *		38.8		31.1		5.8	
**Western Pacific Region (WPRO)**								
**Laos**	**2012**	10,549	47.2	**↓**	6.2	**↑**	1.9	**↑**
	**2017**	8958	34.6		10.2		2.3	
	2030 *		25.4		16.8		2.8	

Where ↓ = projected malnutrition prevalence will be expected to meet GNT by 2030; ↓ = projected malnutrition prevalence will be down-trending but will likely be above the defined GNTs in 2030; ↑ = projected malnutrition prevalence will likely be above the defined GNTs; and ↑ = projected malnutrition prevalence will likely increase but would remain under the defined GNTs. * = projected prevalence of each form of malnutrition for the year 2030 was calculated based on baseline and current prevalence estimates.

### 3.2. Baseline, Current and Scenario-Based Projections of Various Forms of Malnutrition Including CFM Among Children Under Five Across LMICs

Table 2 presented the prevalence of standalone forms of malnutrition (stunting, wasting and obesity) and the CFM, i.e., CWS and CSO among children under five years of 48 LMICs at an individual level.

**Figure 2:**
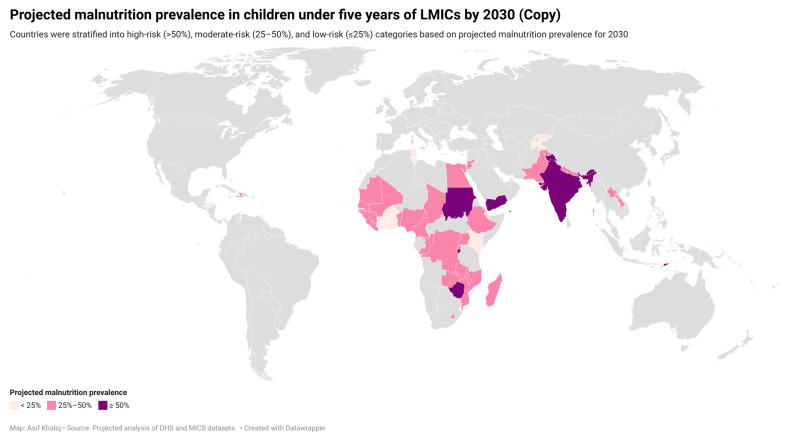
Projected prevalence of malnutrition in children of LMICs by 2030.

At baseline more than half of the children under five were malnourished in 14 countries (Bangladesh, Benin, Burundi, Ethiopia, India, Laos, Madagascar, Malawi, Nepal, Nigeria, Pakistan, Rwanda, Timor-Leste and Yemen). Pediatric malnutrition exceeded 50% in four countries (Burundi, India, Timor-Leste, and Yemen) in the current surveys. In projections for 2030, it is expected that more than half of the children of Sudan and Zimbabwe will also have malnutrition in addition to Burundi, India, Timor-Leste, and Yemen. Considering the projected malnutrition prevalence, the countries were grouped as high-risk (>50%), moderate-risk (25 to 50%) and low-risk countries (≤25%) (Figure 2).

Stunting was the most prevalent form of malnutrition. By 2030, most of the LMICs would be expected to control the raising prevalence of stunting despite its high burden. However, the burden of stunting in Burundi, DRC, Sudan, Yemen, and Zimbabwe would remain high, where one out of every three children below the age of five would be at risk of stunting. By 2030, the projected prevalence of wasting would be expected to increase to over 10% in eight countries: Chad, Egypt, Ethiopia, India, Laos, Mauritania, Senegal, and Timor-Leste. In Egypt, wasting would be expected to cross the prevalence of stunting, which is the most prevalent and chronic form of malnutrition. However, in Jordan and Palestine, childhood obesity is projected to be more prevalent than other forms of malnutrition.

Alongside standalone forms of malnutrition, CFM, specifically the CWS and CSO, was observed in 47 countries, with Kyrgyzstan as the only exception. The prevalence of CSO would be expected to rise in eight countries by 2030, notably observed in Cameron, Ethiopia, Guinea, India, Jordan, Liberia, Palestine, and Timor-Leste. While the prevalence of CWS would be expected to rise in 2030 in 13 countries. These would be Burundi, Chad, Congo, DRC, Egypt, Eswatini, Ethiopia, Laos, Mauritania, Senegal, Sudan, Timor-Leste, and Yemen.

**Table 2:** Scenario-based projections of standalone and coexisting forms of malnutrition to 2030 among children under five across LMICs.

	Survey Period	Malnutrition	Stunting	Wasting	Obesity	CSO	CWS
		Prevalence	Prevalence	Prevalence	Prevalence	Prevalence	Prevalence
**African Region (AFRO)**							
**Benin**	**2012**	62.7	27.6	13.3	4.8	12.8	4.2
	**2018**	38.5	29.4	4.6	1.2	0.7	2.6
	2030 *	34.8	31.3	1.6	0.3	0.0	1.6
**Burundi**	**2010**	59.9	49.2	3.3	0.9	2	4.5
	**2016**	58.6	49.8	2.6	0.8	0.8	4.6
	2030 *	58.1	50.4	2.0	0.7	0.3	4.7
**Burkina Faso**	**2014**	47.8	28.5	11.5	1.1	1.4	5.3
	**2021**	31.7	18.6	8.5	0.8	0.8	3
	2030 *	21.2	12.1	6.3	0.6	0.5	1.7
**Cameron**	**2011**	41.1	27.4	4.5	3.9	2.6	2.7
	**2022**	39.6	23.4	3.7	6.7	4.2	1.6
	2030 *	42.2	20.0	3.0	11.5	6.8	0.9
**Chad**	**2010**	49.9	33.8	9.8	0	0	6.3
	**2019**	44.3	24.2	12.4	0	0	7.7
	2030 *	42.4	17.3	15.7	0	0	9.4
**Comoros**	**2012**	43.6	21.4	10.8	4.2	4.6	2.6
	**2022**	20.9	16.1	4	0	0	0.8
	2030 *	13.8	12.1	1.5	0	0	0.2
**Congo**	**2012**	35.4	24.7	5.4	1.9	1.5	1.9
	**2015**	31.7	23.6	6.1	0	0	2
	2030 *	31.5	22.5	6.9	0	0	2.1
**Côte d’Ivoire**	**2012**	39.1	27	6.4	1.6	1.4	2.7
	**2021**	27	18.8	6	0	0	2.2
	2030 *	20.5	13.1	5.6	0	0	1.8
**DRC**	**2010**	46.4	38.3	5.4	0	0	2.7
	**2018**	47.5	40.4	4.3	0	0	2.8
	2030 *	48.9	42.6	3.4	0	0	2.9
**Eswatini**	**2010**	30.7	29.9	0.5	0	0	0.3
	**2022**	21.6	19.8	1.4	0	0	0.4
	2030 *	17.5	13.1	3.9	0	0	0.5
**Ethiopia**	**2011**	53.9	38.6	8.1	0.9	0.9	5.4
	**2016**	47.9	31.9	9.2	1.2	1	4.6
	2030 *	43.4	26.4	10.4	1.6	1.1	3.9
**Gambia**	**2013**	38.2	21.9	10	1.1	1.7	3.5
	**2020**	26.5	17.6	5.3	1.5	0.3	1.8
	2030 *	19.9	14.1	2.8	2.0	0.1	0.9
**Ghana**	**2014**	39.8	23.8	8.8	2.6	2.3	2.3
	**2022**	26.7	17.2	5.3	1.5	0.4	2.3
	2030 *	18.9	12.4	3.2	0.9	0.1	2.3
**Guinea**	**2012**	42.6	27.1	8.5	1.9	1.7	3.4
	**2018**	42.1	26.1	7.9	2.2	3.3	2.6
	2030 *	43.3	25.1	7.3	2.5	6.4	2.0
**Guinea-Bissau**	**2014**	30.4	24.6	3.7	0	0	2.1
	**2019**	30	25.1	3.1	0	0	1.8
	2030 *	29.7	25.6	2.6	0	0	1.5
**Kenya**	**2014**	44.2	29.3	6.9	2.5	2.7	2.8
	**2022**	27.3	15.5	6.8	2.1	0.6	2.3
	2030 *	18.7	8.2	6.7	1.8	0.1	1.9
**Lesotho**	**2014**	49	35.5	4.4	4.1	3.3	1.7
	**2024**	44.5	34.2	1.9	4.2	2.5	1.7
	2030 *	41.6	32.9	0.8	4.3	1.9	1.7
**Liberia**	**2013**	40	28.2	6.3	1.6	1.4	2.5
	**2020**	39.8	29	4.1	2.6	1.7	2.4
	2030 *	41.1	29.8	2.7	4.2	2.1	2.3
**Madagascar**	**2009**	53.9	37.8	3.9	1.1	4.1	7
	**2021**	45.9	34.9	5.4	0.9	1	3.7
	2030 *	42.6	32.2	7.5	0.7	0.2	2.0
**Malawi**	**2010**	54	39.9	3.9	2.9	5.1	2.2
	**2016**	41.8	32.4	3.3	2.4	2	1.7
	2030 *	33.2	26.3	2.8	2.0	0.8	1.3
**Mali**	**2013**	49.1	32.6	9.6	0.8	1.4	4.7
	**2018**	36.7	23.1	7.8	1	1.2	3.6
	2030 *	27.8	16.4	6.3	1.3	1.0	2.8
**Mauritania**	**2011**	36.3	22.9	9.3	0	0	4.1
	**2015**	36.3	21.5	10.5	0	0	4.3
	2030 *	36.6	20.2	11.9	0	0	4.5
**Mozambique**	**2011**	48.6	33.6	4.9	3.4	4.5	2.2
	**2023**	39.2	30.5	3.1	2.2	1.4	2
	2030 *	33.3	27.7	2.0	1.4	0.4	1.8
**Nigeria**	**2013**	57.4	32.4	11	3.2	5.9	4.9
	**2018**	43.2	32.5	4.8	1.2	0.8	3.9
	2030 *	38.4	32.6	2.1	0.5	0.1	3.1
**Rwanda**	**2013**	50.6	38.9	2.7	3.4	3.6	2
	**2020**	40.4	31.6	2	3.7	2	1.1
	2030 *	32.9	25.7	1.5	4.0	1.1	0.6
**Sao Tome and Principe**	**2014**	19.9	15.8	2.9	0	0	1.2
	**2019**	16.7	12.5	3.5	0	0	0.7
	2030 *	14.5	9.9	4.2	0	0	0.4
**Senegal**	**2011**	31.2	18.1	8.6	1.1	0.3	3.1
	**2023**	30.6	16.9	9.3	0.8	0.2	3.4
	2030 *	30.3	15.8	10.1	0.6	0.1	3.7
**Sierra Leone**	**2013**	49	27.1	9.6	4.2	5.2	2.9
	**2019**	38.9	26.7	4.7	2.6	2.1	2.8
	2030 *	33.7	26.3	2.3	1.6	0.8	2.7
**Togo**	**2010**	36.3	30.7	2.6	0	0	3
	**2017**	28.4	22.6	3.8	0	0	2
	2030 *	23.5	16.6	5.6	0	0	1.3
**Uganda**	**2011**	40.5	29.1	4.8	2.2	1.8	2.6
	**2019**	36	26.7	3.9	2.6	1.3	1.5
	2030 *	32.6	24.5	3.2	3.1	0.9	0.9
**Zambia**	**2013**	48.7	35	5.8	2.5	3.3	2.1
	**2018**	42.5	31.3	4.4	2.6	2.6	1.6
	2030 *	37.2	28.0	3.3	2.7	2.0	1.2
**Zimbabwe**	**2011**	39.6	28.8	3.8	3.2	2.4	1.4
	**2015**	44.8	32.9	4.3	3.9	2.4	1.3
	2030 *	50.9	37.6	4.9	4.8	2.4	1.2
**American Region (AMRO)**							
**Haiti**	**2012**	30.9	20.5	4.9	2.3	1.3	1.9
	**2017**	29.4	20.3	4.2	2.3	1.2	1.4
	2030 *	28.1	20.1	3.6	2.3	1.1	1.0
**Honduras**	**2012**	33.5	25	2.3	4.2	0.8	1.2
	**2019**	21.1	19.3	1.3	0	0	0.5
	2030 *	15.8	14.9	0.7	0	0	0.2
**Eastern Mediterranean Region (EMRO)**							
**Egypt**	**2008**	45	18.3	7.5	8.1	10.2	0.9
	**2014**	38.1	13.6	10.8	6.3	6.4	1
	2030 *	35.7	10.1	15.6	4.9	4.0	1.1
**Jordan**	**2012**	15	7.7	2.1	4.1	0.9	0.2
	**2023**	18.5	6.9	2.3	6.9	2.3	0.1
	2030 *	26.3	6.2	2.5	11.6	5.9	0.1
**Pakistan**	**2012**	52.8	35.4	5.9	1.7	5.2	4.6
	**2017**	44.9	33.8	5.1	1.3	1.8	2.9
	2030 *	40.1	32.3	4.4	1.0	0.6	1.8
**Palestine**	**2010**	17.8	9.2	2.9	3.9	1.5	0.3
	**2020**	18.3	7.1	1.4	8.1	1.6	0.1
	2030 *	24.7	5.5	0.7	16.8	1.7	0.0
**Sudan**	**2010**	45.7	29.5	9.9	0	0	6.3
	**2014**	48.5	33.1	8.7	0	0	6.7
	2030 *	51.8	37.1	7.6	0	0	7.1
**Tunisia**	**2011**	12.8	10.3	2.3	0	0	0.2
	**2023**	15.5	12.4	2.9	0	0	0.2
	2030 *	18.8	14.9	3.7	0	0	0.2
**Yemen**	**2013**	58.2	38.4	10.7	0.7	1.4	7
	**2023**	58.3	39.9	9.5	0	0	8.9
	2030 *	61.2	41.5	8.4	0	0	11.3
**European Region (EURO)**							
**Kyrgyzstan**	**2014**	15.2	13	2	0	0	0.2
	**2018**	12.8	10.8	2	0	0	0
	2030 *	11	9.0	2.0	0	0	0
**Tajikistan**	**2012**	38.6	21.4	10	2.4	2.9	1.9
	**2017**	28.7	16.7	6.9	2.2	1.7	1.2
	2030 *	21.6	13.0	4.8	2.0	1.0	0.8
**South-East Asian Region (SEARO)**							
**Bangladesh**	**2011**	51.9	33.1	9.5	0.9	0.7	7.7
	**2022**	34.7	20	9.1	1.1	0.4	4.1
	2030 *	24.5	12.1	8.7	1.3	0.2	2.2
**India**	**2015**	55	30.8	14.7	0.9	1.5	7.1
	**2021**	52.5	29	14.4	1.3	2.3	5.5
	2030 *	51.1	27.3	14.1	1.9	3.5	4.3
**Nepal**	**2011**	50.7	37.3	7.2	0.6	0.6	5
	**2022**	35	24.7	5.6	1	0.2	3.5
	2030 *	25.1	16.4	4.4	1.7	0.1	2.5
**Timor-Leste**	**2010**	71.7	46.5	12.9	0.9	3.4	8
	**2016**	65.6	35.1	16.8	1.4	3.6	8.7
	2030 *	63.9	26.5	21.9	2.2	3.8	9.5
**Western Pacific Region (WPRO)**							
**Laos**	**2012**	51.3	43.2	3.4	0.7	1.2	2.8
	**2017**	42.7	30.2	6.9	1.2	1.2	3.2
	2030 *	42.1	21.1	14.0	2.1	1.2	3.7

Where malnutrition = sum of all forms of standalone (stunting, wasting, obesity) and CFM; CWS = coexistence of wasting with stunting; CSO = coexistence of stunting with obesity; and * = projected prevalence of each form of malnutrition for the year 2030 was calculated based on baseline and current prevalence estimates.

### 3.3. Determinants of Standalone and Coexisting Forms of Malnutrition Among Children Under Five Across LMICs

Table 3 presents the inferential analysis, in which the relationship of various forms of malnutrition with individual, household, regional and community and temporal factors were assessed via the Generalized Linear Model (GzLM) framework with binomial logistic regression.

We found significantly higher odds of stunting by 15% (14% to 15%) with each increasing month of age among children between the ranges of 0 to 59 months age. On the other hand, the odds of wasting and obesity decreased by 14% (13% to 14%) and 16% (15% to 17%) with each increasing month of age among children between the ranges of 0 to 59 months age. The odds of CWS increased by 1.03 (1.02 to 1.03) and the CSO decreased by 0.83 (0.82 to 0.84) with each increasing month of age among children between the ranges of 0 to 59 months age. Compared to female children, the male children are at significantly increased odds of acquiring stunting, wasting and obesity of 1.14 (1.13 to 1.15), 1.10 (1.08 to 1.11), 1.13 (1.09 to 1.16), respectively. Similarly, the CWS is associated with an increased odds of 1.46 (1.43 to 1.48) and the CSO is significantly associated with an increased odds of 1.12 (1.08 to 1.15) among male children between the ranges of 0 to 59 months age.

Compared to parents who have acquired higher education, the odds of stunting and wasting were increased by approximately 1- to 2-fold among parents who have acquired less education or no education. Lower level of parents’ education was significantly associated with the lower odds of having children with obesity, ranging from 21% to 29%. The odds of CWS increased by approximately 1.5- to 3-fold among parents who have acquired less education or no education compared to parents who have acquired higher education. The CSO increased the odds to 1.41 (1.33 to 1.50) among parents who have no education. On contrary, the odds of the CSO decreased to 0.89 (0.84 to 0.94) among parents who have acquired secondary education. However, no association was observed with the CSO among parents who have acquired primary education when compared with parents having higher education. Overall, the parental educational level is significantly associated with increased odds of malnutrition among children under five across LMICs.

When compared with the richest wealth index, the odds of developing stunting and wasting increased by approximately 1–2-fold among children belonging to the poorest wealth index. The odds of obesity with each wealth index, namely, from poor to richer, significantly decreased by 18% to 38% when compared with the children belonging to the richest wealth index. The odds of CWS were 2.5-fold higher among children belonging to the poorest wealth index as compared to richest wealth index.

We found significantly higher odds of stunting and wasting of 1.19 (1.17 to 1.21) and 1.04 (1.02 to 1.06), respectively, among the children residents of rural areas as compared to the children residents of rural area. On the other hand, the odds of obesity decreased to 0.99 (0.95 to 1.03) among the children residents of rural areas. The odds of CWS increased by 1.14 (1.11 to 1.17) and the CSO increased to 1.13 (1.08 to 1.17) among the children’s residents of rural areas. When compared with WPRO the odds of stunting decreased to 0.85 (0.83 to 0.87), 0.52 (0.50 to 0.54), 0.83 (0.81 to 0.86), and 0.49 (0.47 to 0.51) in children belonging to the regions of AFRO, AMRO, EMRO and EURO. However, the odds of stunting increased to 30% (27% to 34%) in children belonging to the region of SEARO. There were approximately 1–3-fold higher odds of wasting in the regions of AFRO, EMRO, EURO and SEARO when compared with WPRO. On the contrary, there were lower odds of wasting in the regions of AMRO of 0.59 (0.46 to 0.75). Compared with WPRO, across all five LMICs, the odds of obesity increased by approximately 3- to 4.5-fold. CWS was associated with odds increased by approximately 1- to 3-fold in the regions of AFRO, EMRO, and SEARO. The CWS was significantly lower, with odds of 0.32 (0.28 to 0.36) and 0.36 (0.30 to 0.43) in the regions of AMRO and EURO. The CSO is associated with the increased odds of approximately 2- to 5-fold in the regions of AFRO, EMRO, EURO and SEARO. However, the CSO is associated with the lower odds of 0.49 (0.24 to 1.04).

The odds of stunting, wasting and obesity decreased to approximately 3% with an increased passing year. Similarly, the odds of CWS decreased to 0.97 (0.97 to 0.97) and the CSO decreased to 0.94 (0.94 to 0.94) with an increased passing year among children under five across LMICs (Table 3).

### 3.4. Interaction Effect Between Survey Year and Selected Covariates of Standalone and Coexisting Forms of Malnutrition Among Children Under Five Across LMICs

Table 4 presented the inferential analysis, in which the interaction effects between survey year and selected covariates on standalone and coexisting forms of malnutrition among children under five in low- and middle-income countries (LMICs) were assessed via binomial logistic regression with interaction.

Across the two survey periods, with each increasing month in age there was a 1% reduction in the odds of stunting among children under five in LMICs. Conversely, each additional month of children age was associated with 1% increase in the odds of wasting. There was no significant difference observed in the odds of child’s obesity trend over time. With each increasing month in age, there was a slight but significant reduction in the odds of CWS (OR = 0.99; CI: 0.99–0.99) and the CSO (OR = 0.98; CI: 0.97–0.98).

The yearly change in stunting, wasting and obesity was not associated with children’s sex. The CSO was increased in the odds of 1.01 (1.00 to 1.02) among male children as compared to female counterparts.

The odds of stunting and wasting showed a small but statistically significant reduction of approximately 1% among children whose parents had lower levels of education. The odds of obesity were reduced by approximately 8% (7% to 10%) among children of parents who had no formal education. There was a significant reduction in the odds of CWS of approximately 2% to 7% among children whose parents had lower levels of education. And the CSO was reduced by approximately 2% to 8% among children whose parents had primary level of education and no formal education. Conversely, the odds of CSO increased to approximately 2% (1% to 4%) among children whose parents had a secondary level of education.

The yearly change in stunting showed a small but statistically significant reduction of approximately 2–3% among children belonging to the richer, middle, poorer, and poorest wealth indices compared with those in the richest wealth index. Across the two survey periods, the odds of wasting increased slightly among children in the poorest wealth index (OR = 1.01; 95% CI: 1.00–1.02). No statistically significant association was observed between wealth status and wasting among children in the middle and richest wealth indices (OR = 1.00; 95% CI: 0.99–1.01). In contrast, the odds of obesity were reduced by approximately 2% (CI: 1–3%) among children belonging to the poorest wealth index. The risk of stunting, wasting and obesity was not associated with place of residence across the two survey periods. The odds of CSO increased to approximately 1% to 2% among children belonging to the poorest to middle wealth index.

Children in the AFRO and EURO regions had a 2–3% lower risk of stunting compared with the WPRO region. Children of AMRO and EMRO are at an increased risk of stunting of approximately 2% and 7%, respectively. The risk of wasting for the children in the AFRO and AMRO regions is statistically reduced by 0.95 (0.94 to 0.97) and 0.97 (0.95 to 0.99), respectively. The odds of wasting were reduced by approximately 11% (CI: 8–12%) among children of EURO across the two survey periods. Compared with WPRO, across all four regions namely, AFRO, EMRO, EURO and SERO LMICs, the odds of obesity were increasing with the odds of obesity ranging from 6% to 13%, while no significant association was observed for AMRO 1.00 (0.98 to 1.02). The odds of CWS were reduced in the region of AFRO, 0.98 (0.96 to 0.98), and SEARO, 0.98 (0.96 to 0.99), as compared to the region of WPRO. The odds of CWS were significantly increased in the region of AMRO, 1.02 (1.00 to 1.05), and EMRO, 1.08 (1.06 to 1.09). The CSO among children was significantly reduced to 14% to 36% across all five LMICs, as compared to region of WPRO.

## 4. Discussion

This study measured the projected trajectories of stunting, wasting, obesity, and CFM among children under five across 48 LMICs to 2030. The findings revealed uneven malnutrition reduction across different regions and countries, although child mortality attributable to malnutrition has declined globally due to improvements in healthcare delivery [24,25]. The study reported that the majority of the countries are projected to meet one or more GNTs by 2030, while most remain off-track to fully achieve the nutrition commitments under the SDGs. This is because the proposed GNTs in alignment with SDGs are more ambitious compared to the GNT recommended by the World Health Assembly. The GNTs for stunting, wasting and obesity were 40% reduction in stunting from baseline, wasting below 5% and halting the rise in obesity, while the targets under SDGs were 50% stunting reduction from baseline and below 3% for wasting and obesity by 2030 [26]. The dominant pattern across indicators is a widening divergence between countries with stable systems and those affected by conflict, climate shocks, or weak governance [27,28].

Among various forms of pediatric malnutrition, stunting, which is also known as chronic undernutrition, is responsive to long-term, multisectoral strategies. Sustained investments in maternal health, sanitation, education, and primary care have remarkably reduced the rising burden of stunting in most on-track countries [29,30]. However, projections reveal important fragility, because the inequality gaps between the on-track and off-track LMICS narrowed temporarily between the baseline and current surveys but are projected to widen again by 2030, i.e., stunting is expected to remain above 40% in Burundi, Mauritania, and Yemen, with Sudan and Zimbabwe at risk of joining this group (Table 1). These trajectories suggest that stunting reduction requires sustained structural transformation [31,32]. Unlike stunting, wasting responds rapidly to acute shocks, such as conflict, drought, displacement, food price volatility, and infectious disease outbreaks [27,28]. By the year 2030, more than half of LMICs are projected to meet ≤5% threshold GNT for wasting. Furthermore, the on-track countries will show gradual improvements for wasting reduction, while the off-track countries are expected to experience rising prevalence. This divergence mirrors patterns described by the Lancet Commission on the Future of Health, where improvements in stable systems coexist with deterioration in fragile states [33]. Countries such as Sudan, Yemen, Zimbabwe, Chad, and Timor-Leste demonstrate particularly concerning trajectories (Table 1). Even small absolute reductions in wasting among on-track countries represent meaningful system-level strengthening in acute malnutrition management. In contrast, minimal change or rising prevalence in fragile settings reflects structural vulnerability and repeated humanitarian shocks [27,28]. By 2030, wasting inequality between country groups is projected not merely to return to baseline levels but to surpass them.

The present study does not suggest a significant increasing trend of obesity in children over time. That is contrary to the global evidence proposed by the UNICEF, WHO and World Bank of the continual increasing trend of obesity among children belonging to LMICs [3]. Nevertheless, obesity in children under five years remains an emerging nutritional challenge that follows a structurally different but equally concerning trajectory. The prevalence of obesity in more than half of LMICs remains below the 5% threshold; however, projections indicate that by 2030, several countries across Asia, Africa, and the Middle East are likely to experience increasing trends, particularly those still burdened by undernutrition (Table 1 and Table 2). The discrepancy in present research findings might be attributed to the dynamic heterogeneous nature and persistent changing pattern of obesity in children belonging to LMICs that can erroneously mask the population data [34]. The rising prevalence of obesity coexists in certain countries, such as Jordan, Palestine, Timor-Leste, and Zimbabwe, with undernutrition (stunting or wasting or both) (Table 1), thereby indicating the double burden of malnutrition, a phenomenon in which ultra-processed food penetration drives over nutrition even within populations still struggling with acute undernutrition [35]. This reveals bidirectional movement between the on-track and off-track countries by 2030. The on-track countries show modest but consistent declines due to policy interventions targeting obesogenic environments [36]. Conversely, the off-track countries are projected to experience further increases driven by rapid dietary transitions, ultra-processed food penetration, urbanization, and weak regulatory frameworks [37,38]. Overall, these patterns may reflect a systemic transition of food and environment rather than short-term fluctuations, hence regarding obesity a transitional and multifaceted problem in LMICs [35].

CFM (CWS and CSO) was identified in nearly all LMICs, underscoring the complexity of current nutrition landscapes. Despite its global prevalence, still the targets for the reduction in CFM and its various forms are not yet defined by any local and international body. By 2030, the prevalence of both CWS and CSO is projected to rise. Countries like Ethiopia, India, Laos, and Timor-Leste indicate rising projection of CWS by 2030 (Table 2), and this depicts a complex relation between chronic deprivation and acute shocks [27,28]. However, an escalated projection of CSO in several conflict-affected countries including Yemen and Sudan (Table 2) reflects calorie-dense but micronutrient-poor diets, humanitarian food assistance dependency, and rapid market shifts toward processed foods [35,37].

Countries with deteriorating trajectories represent critical regional hotspots. Five countries are projected to have stunting prevalence persist above 40% by 2030: Burundi (54.1%), Mauritania (49.4%), Sudan (61.3%), Yemen (51.4%), and Zimbabwe (56.6%). Wasting remains the most challenging indicator across LMICs, with 33 countries currently exceeding the 5% GNT threshold and 22 projected to continue exceeding this threshold by 2030. Childhood obesity is rising globally, with 14 countries expected to exceed the 2030 threshold. We observed concerning levels of CSW, exceeding 4% in Ethiopia (20.3%), Cameroon (5.9%), Laos (5.5%), and India (4.5%). CSW represents the most lethal form of malnutrition, with mortality risk twelve times higher than well-nourished children. Moreover, the escalated projection of CSO to over 5% in Timor-Leste (10.1%), Chad (9.8%), and other conflict-affected settings highlight the emerging double burden in fragile and transitioning contexts [27,28,37] (Table 2).

Across regions, high-risk countries share common drivers: conflict, climate shocks, weak health systems, and structural poverty. The AFRO region remains the epicenter of the global malnutrition crisis with several countries classified either as in the danger zone or critical hotspots requiring emergency response. In AFRO region, Burundi, Mali, Mauritania, and Zimbabwe continue to experience stunting likely due to suboptimal exclusive breastfeeding, limited dietary diversity, infectious disease burden and limited community-based nutrition programs including poor antenatal care [24,29,39,40]. Ethiopia demonstrated an unexpected CWS crisis (projected 20.3%) secondary to the compounded effects of the Tigray conflict, with severe drought, locust attacks and the COVID-19 pandemic. Benin presents an unusual trajectory of rising projected stunting (33.4%) despite a marked decline in wasting (from 13.3% to 0.6%), suggesting improvement in acute malnutrition management but persistent chronic determinants. In EMRO, Sudan and Yemen showed protracted humanitarian catastrophes with persistently high stunting and CSO, likely linked to prolonged nutritionally inadequate food assistance in the conflict-affected countries [41]. While Jordan and Palestine illustrate emerging childhood obesity secondary to nutrition transition and profound dietary shifts [42]. In SEARO, India and Timor-Leste showed slow progress to reach the GNTs due to poor feeding practices, lack of sanitary measures, and fragile infrastructure [43]. Similarly, in the WPRO region, Laos presents catastrophic projection of wasting (Table 2). Collectively, these countries represent danger zones where chronic deprivation intersects with acute shocks, producing persistent and, in some settings, worsening malnutrition.

In contrast, certain countries from each region achieve the GNTs and have successfully reduced the rising burden of various forms of malnutrition, including CFM. In the AFRO region, Chad, Congo, Ghana and Rwanda achieved substantial stunting reduction through coordinated national nutrition mechanisms [24,44]. Despite tremendous reduction in stunting, still Chad illustrates a complex pattern of stunting alongside with wasting (CWS) and obesity (CSO), thereby reflecting the humanitarian-induced obesity among the stunting children of Chad [45]. In SEARO, Bangladesh is projected to improve pediatric nutritional status by incorporating multisectoral improvements: sanitation, dietary diversity, women’s empowerment, and health service expansion [46]. In AMRO, Honduras approaches near-elimination thresholds, whereas Haiti showed persistent challenge due to political instability and natural disaster. Within EURO, Tajikistan and Kyrgyzstan combatted the rising malnutrition prevalence by improving the health infrastructure and social protection systems (Table 2).

Child age and sex emerged as fundamental biological determinants of malnutrition. Each additional year increased in child age increases the stunting odds by 15% while decreasing wasting (14%) and obesity (16%). Different studies demonstrated that acute malnutrition peaks between 6 and 18 months when complementary feeding is introduced and infectious disease exposure intensifies, while the chronic growth faltering accumulates thereafter [39,47]. Between male and female children, higher odds of undernutrition, particularly for CWS, were observed in male children and this is in line with recent systematic review evidence [48].

Maternal education was the strongest and most consistent predictor across all forms of malnutrition. Children of mothers with no education had 2.19-fold higher odds of stunting compared with those whose mothers had higher education, and similar gradients were observed for wasting, obesity, and CFM. These findings are consistent with a 2024 meta-analysis across 35 studies demonstrating a robust inverse association between maternal education and child undernutrition [49]. Moreover, maternal education also improved the knowledge and practices for appropriate feeding practices and thereby helped to reduced malnutrition [50]. Similarly, children from the poorest wealth quintile exhibited 2.00-fold higher odds of stunting compared to the richest quintile. Analysis of DHS data from 24 LMICs (2017–2022) confirms that child undernutrition remains statistically significant with wealth-related inequality across country income categories [51]. Children of rural residence have high risks of nutritional adversities (malnutrition), highlighting structural disadvantages in healthcare access, WASH infrastructure, and food systems [52].

Interaction analyses revealed that structural inequalities are narrowing too slowly to meet 2030 targets. With time, the poverty gap in stunting is closing by only 2% annually, implying that parity between poorest and richest quintiles would take approximately five decades without any major policy shifts. Similarly, the rural–urban differential narrowed by just 1% per year, indicating persistent geographic disadvantage. Over time, the difference in stunting between younger and older children has reduced by about 1% each year. A similar small improvement was seen for CWS. This suggests that nutrition and child health programs may be gradually improving conditions in early childhood, although the progress is modest.

In contrast, the difference between boys and girls has remained mostly unchanged for most forms of malnutrition. However, for CSO, the gap has slowly widened, with boys becoming slightly more affected over time. This trend may reflect changing diets and broader nutrition transition patterns [53]. Regionally, EMRO demonstrated worsening trends, with stunting and obesity increasing by approximately 7% annually relative to other regions, whereas AFRO exhibited faster stunting reduction (≈2% annual acceleration). Overall, the year interaction terms capture the cumulative impact of nutrition-specific and nutrition-sensitive interventions between 2012 and 2023, indicating modest progress but persistent inequities unlikely to resolve under business-as-usual trajectories [3].

### 4.1. Strengths and Limitation

This study draws on a very large, multi-country sample of more than a million children under 5 years from 48 LMICs, using DHS and MICS that employ multistage stratified cluster sampling, standardized questionnaires, experienced field workers, and validated anthropometric protocols [37,54]. Uniform procedures for data screening, cleaning, coding, and categorization, along with sample-weight adjustments and harmonization of key variables, enhanced the validity, precision and comparability of estimates across countries over time. By triangulating the DHS and MICS datasets and standardizing variable definitions, the analysis achieves broader geographical coverage and more consistent measures than studies restricted to a single survey platform. The use of regression models that include interaction terms between the survey period and key covariates allows assessment of how social and regional gradients in malnutrition have evolved over time, rather than relying solely on crude prevalence differences. Despite the large sample size and inclusion of many LMICs, the findings cannot be considered fully representative of all regions because of under-representation of WPRO, EURO, and AMRO region and some parts of EMRO and SEARO region. Several countries that are no longer classified as LMICs were retained because they received development assistance or were LMICs at the time of data collection, which may complicate the strict alignment with current World Bank Income groupings [23,55]. The pooled data estimates and projections may not reflect the effects of recent conflicts, climate-related disasters, economic crises, or pandemics. Exclusion of around 10–15% of children due to incomplete anthropometry or outliers, while undoubtedly necessary for data quality, may lead to underestimations of malnutrition in settings with poorer measurement. Furthermore, the survey sampling frames primarily target women of reproductive age, with children under 5 nested within these samples, which can distort the representativeness of the pediatric population. Finally, the malnutrition projections measured in this study relied on the past trajectories; therefore, they must be interpreted as indicative rather than an exact forecast.

### 4.2. Policy Implications

Addressing pediatric malnutrition through GNTs remains a critical global priority. However, progress in many LMICs continues to lag behind these targets, partly because current benchmarks are defined by individual anthropometric indicators rather than by the nutritional status of the child as a whole. The presence of one form of malnutrition does not preclude the coexistence of others, highlighting a key limitation of standalone indicator–based approaches. There is a clear need to expand national and regional nutrition surveillance beyond single indicators to systematically capture CFM. This would enable earlier identification and timely management of children affected by multiple nutritional deficits. In addition, redefining the GNTs to include individual-level benchmarks alongside national-level goals could help to reduce the rising burden of malnutrition, including CFM. Such an approach would also support policymakers and program managers in designing integrated strategies that simultaneously address undernutrition and overnutrition at the individual, community, and national levels.

### 4.3. Future Directions

Survey frequency across LMICs remains uneven, limiting the ability to comprehensively track nutritional trends over time. More regular and systematically scheduled surveys would allow a clearer understanding of evolving nutrition profiles. DHS and MICS implementing agencies should prioritize greater inclusion of underrepresented regions particularly AMRO, EURO, and WPRO region to ensure more equitable geographic coverage. Integrating nutrition survey data with climatic, environmental, and humanitarian indicators would yield deeper insights into external determinants influencing the national nutrition targets and prevalence of each form of malnutrition including CFM. Furthermore, the adoption of longitudinal study designs including interventional studies would strengthen the identification of context-specific, underlying, and currently underexplored factors contributing to meeting the national nutrition targets by combating malnutrition including CFM in children.

## 5. Conclusions

This study demonstrates the need to incorporate CFM tracking and measurement alongside traditional nutritional indicators. Many LMICs are not on track to meet the 2030 child-growth targets. While some countries show progress, the overall pace of improvement is slow and marked by persistent socioeconomic and regional inequalities. Without accelerated and targeted action, the burden of both standalone and CFM will remain substantial. This study highlights an urgency for nutritional interventions, health promotion, and healthy food subsidies particularly among children under five years of age. Governments, global health partners, and policy makers should integrate CFM into surveillance and guidelines and support research into subclinical cases and intervention effects to achieve meaningful reductions by prioritizing multisectoral strategies.

## Figures and Tables

**Table 3:** Cross-survey temporal assessment of standalone and coexisting forms of malnutrition among children under five across LMICs.

Covariates	Stunting OR (95% CI)	Wasting OR (95% CI)	Obesity OR (95% CI)	CWS OR (95% CI)	CSO OR (95% CI)
**Individual Factors**					
Age of child (in year)	1.15 (1.14 to 1.15) *	0.86 (0.86 to 0.87) *	0.84 (0.83 to 0.85) *	1.03 (1.02 to 1.03) *	0.83 (0.82 to 0.84) *
Child sex:					
Female (reference)	Ref	Ref	Ref	Ref	Ref
Male	1.14 (1.13 to 1.15) *	1.10 (1.08 to 1.11) *	1.13 (1.09 to 1.16) *	1.46 (1.43 to 1.48) *	1.12 (1.08 to 1.15) *
**Household Factors**					
Education:					
None	2.19 (2.14 to 2.23) *	1.54 (1.50 to 1.59) *	0.79 (0.74 to 0.84) *	2.94 (2.79 to 3.09) *	1.41 (1.33 to 1.50) *
Primary	1.99 (1.94 to 2.03) *	1.19 (1.15 to 1.22) *	0.74 (0.69 to 0.78) *	2.21 (2.10 to 2.33) *	1.03 (0.96 to 1.09)
Secondary	1.41 (1.39 to 1.44) *	1.10 (1.07 to 1.12) *	0.71 (0.68 to 0.75) *	1.51 (1.44 to 1.59) *	0.89 (0.84 to 0.94) *
Higher (reference)	Ref	Ref	Ref	Ref	Ref
Wealth index					
Poorest	2.00 (1.96 to 2.04) *	1.48 (1.26 to 1.73) *	0.62 (0.59 to 0.69) *	2.59 (2.48 to 2.70) *	0.73 (0.44 to 1.21)
Poorer	1.73 (1.71 to 1.77) *	1.24 (1.05 to 1.47) *	0.68 (0.64 to 0.72) *	1.95 (1.87 to 2.04) *	0.64 (0.37 to 1.11)
Middle	1.51 (1.48 to 1.54) *	1.30 (1.10 to 1.54) *	0.79 (0.75 to 0.83) *	1.58 (1.51 to 1.65) *	0.59 (0.33 to 1.05)
Richer	1.27 (1.25 to 1.30) *	1.20 (1.01 to 1.42) *	0.82 (0.78 to 0.86) *	1.29 (1.23 to 1.34) *	0.69 (0.40 to 1.18)
Richest (reference)	Ref	Ref	Ref	Ref	Ref
**Regional and Community Factors**					
Place of residence					
Rural	1.19 (1.17 to 1.21) *	1.04 (1.02 to 1.06) *	0.99 (0.95 to 1.03)	1.14 (1.11 to 1.17) *	1.13 (1.08 to 1.17) *
Urban (reference)	Ref	Ref	Ref	Ref	Ref
Geographical regions					
AFRO	0.85 (0.83 to 0.87) *	1.24 (1.08 to 1.40) *	4.82 (3.99 to 5.84) *	1.05 (0.98 to 1.13)	2.70 (1.89 to 3.86) *
AMRO	0.52 (0.50 to 0.54) *	0.59 (0.46 to 0.75) *	6.00 (4.95 to 7.45) *	0.32 (0.28 to 0.36) *	0.49 (0.24 to 1.04)
EMRO	0.83 (0.81 to 0.86) *	1.73 (1.50 to 1.98) *	6.65 (5.49 to 8.06) *	1.64 (1.51 to 1.76) *	5.13 (3.58 to 7.36) *
EURO	0.49 (0.47 to 0.51) *	1.65 (1.39 to 1.97) *	3.00 (2.39 to 3.78) *	0.36 (0.30 to 0.43) *	3.90 (2.58 to 5.92) *
SEARO	1.30 (1.27 to 1.34) *	3.65 (3.21 to 4.15) *	4.56 (3.76 to 5.51) *	3.27 (3.05 to 3.51) *	5.10 (3.59 to 7.28) *
WPRO (reference)	Ref	Ref	Ref	Ref	Ref
**Temporal Factor**					
Year	0.97 (0.97 to 0.98) *	0.98 (0.98 to 0.98) *	0.98 (0.97 to 0.98) *	0.97 (0.97 to 0.97) *	0.94 (0.94 to 0.94) *

Where CWS = coexistence of wasting with stunting; CSO = coexistence of stunting with obesity; OR = odds ratio; CI = Confidence Interval; Ref = reference category; and * = statistically significant association.

**Table 4:** Interaction effects between survey year and selected covariates on standalone and coexisting forms of malnutrition among children under five in low- and middle-income countries (LMICs).

Covariates	Stunting OR (95% CI)	Wasting OR (95% CI)	Obesity OR (95% CI)	CWS OR (95% CI)	CSO OR (95% CI)
**Individual Factors**					
Year x Age of child (in year)	0.99 (0.99 to 0.99) *	1.01 (1.01 to 1.01) *	1.00 (0.99 to 1.00)	0.99 (0.99 to 0.99) *	0.98 (0.97 to 0.98) *
Child sex x Year					
Female (reference)	Ref	Ref	Ref	Ref	Ref
Male	0.99 (0.99 to 1.01)	1.01 (0.99 to 1.01)	1.00 (0.99 to 1.01)	0.99 (0.99 to 1.00)	1.01 (1.00 to 1.02) *
**Household Factors**					
Education x Year					
None	0.99 (0.99 to 1.00)	0.99 (0.98 to 0.99) *	0.92 (0.90 to 0.93) *	0.95 (0.93 to 0.96) *	0.92 (0.91 to 0.94) *
Primary	0.99 (0.98 to 0.99) *	0.99 (0.98 to 0.99) *	0.99 (0.97 to 1.00)	0.93 (0.92 to 0.95) *	0.98 (0.96 to 0.99) *
Secondary	0.99 (0.98 to 0.99) *	1.00 (0.99 to 1.01)	1.00 (0.99 to 1.01)	0.98 (0.96 to 0.99) *	1.02 (1.01 to 1.04) *
Higher (reference)	Ref	Ref	Ref	Ref	Ref
Wealth index x Year					
Poorest	0.98 (0.97 to 0.98) *	1.01 (1.00 to 1.02) *	0.98 (0.97 to 0.99) *	1.01 (0.99 to 1.05)	1.02 (1.01 to 1.04) *
Poorer	0.98 (0.97 to 0.98) *	1.01 (0.99 to 1.01)	0.99 (0.98 to 1.01)	0.99 (0.99 to 1.00)	1.01 (1.00 to 1.03) *
Middle	0.98 (0.97 to 0.99) *	1.00 (0.99 to 1.01) *	1.00 (0.99 to 1.01)	0.99 (0.99 to 1.00)	1.02 (1.00 to 1.03) *
Richer	0.97 (0.96 to 0.97) *	1.00 (0.99 to 1.01)	0.99 (0.98 to 1.00)	0.99 (0.99 to 1.00)	1.00 (0.98 to 1.02)
Richest (reference)	Ref	Ref	Ref	Ref	Ref
**Regional and Community Factors**					
Place of residence x Year					
Rural	0.99 (0.99 to 1.00)	0.99 (0.99 to 1.00)	0.99 (0.99 to 1.00)	1.88 (1.88 to 1.89) *	1.01 (0.99 to 1.01)
Urban (reference)	Ref	Ref	Ref	Ref	Ref
Geographical regions x Year					
AFRO	0.98 (0.97 to 0.99) *	0.95 (0.94 to 0.97) *	1.10 (1.08 to 1.11) *	0.98 (0.96 to 0.98) *	0.72 (0.67 to 0.76) *
AMRO	1.02 (1.01 to 1.03) *	0.97 (0.95 to 0.99) *	1.00 (0.98 to 1.02) *	1.02 (1.00 to 1.05) *	0.69 (0.64 to 0.75) *
EMRO	1.07 (1.06 to 1.08) *	1.01 (0.99 to 1.02)	1.07 (1.06 to 1.09) *	1.08 (1.06 to 1.09) *	0.71 (0.66 to 0.76) *
EURO	0.97 (0.96 to 0.99) *	0.89 (0.88 to 0.92) *	1.06 (1.03 to 1.10) *	0.99 (0.96 to 1.02)	0.64 (0.59 to 0.71) *
SEARO	1.00 (0.99 to 1.01)	0.99 (0.98 to 1.01)	1.13 (1.11 to 1.14) *	0.98 (0.96 to 0.99) *	0.86 (0.81 to 0.92) *
WPRO (reference)	Ref	Ref	Ref	Ref	Ref

Where x = interaction of each study covariate with survey years for each form of malnutrition CWS = coexistence of wasting with stunting; CSO = coexistence of stunting with obesity; OR = odds ratio; CI = Confidence Interval; Ref = reference category; and * = statistically significant association.

## Data Availability

The data from this study can be retrieved from the DHS program (www.dhsprogram.com) and UNICEF (https://mics.unicef.org/surveys, accessed on 30 October 2025).

## Supplementary Materials

The following supporting information can be downloaded at: https://www.mdpi.com/article/10.3390/nu18071160/s1, File S1: The S1 comprises nine excel sheets presenting baseline and current prevalence estimates, along with 95% confidence intervals, for key malnutrition indicators (stunting, wasting, and obesity) and nutritional outcomes (normal nutritional status, stunting, wasting, obesity, CWS, and CSO) among children under five years of age; File S2: The File S2 includes excel sheets detailing baseline, current, and projected prevalence estimates for the same malnutrition indicators (stunting, wasting and obesity) and nutritional outcomes (stunting, wasting, obesity, normal status, CWS, and CSO) in children under five years. Projections are derived using the Annual Rate of Reduction (ARR) and corresponding prevalence estimates.

## Abbreviations

AFRO	African Region
AMRO/PAHO	Region of the Americas/Pan American Health Organization
ARR	Annual Rate of Change
CFM	Coexisting Forms of Malnutrition
COVID-19	Coronavirus Disease 2019
CSO	Coexistence of Stunting with Obesity
CWS	Coexistence of Wasting with Stunting
DHS	Demographic and Health Surveys
DRC	Democratic Republic of Congo
EMRO	Eastern Mediterranean Region
EURO	European Region
GBD	Global Burden of Disease
GzLM	Generalized Linear Model
GNR	Global Nutritional Report
GNT	Global Nutrition Target
HAZ	Height-for-Age Z-score
JME	Joint Malnutrition Estimates
LICs	Low-Income Countries
LMICs	Low- and Middle-Income Countries
MDGs	Millennium Development Goals
MICS	Multiple Indicator Cluster Surveys
OR	Odds Ratio
PDHS	Pakistan Demographic and Health Surveys
SDGs	Sustainable Development Goals
SEARO	South-East Asia Region
SFM	Standalone Forms of Malnutrition
UHC	Universal Health Coverage
UICs	Upper-Income Countries
UNICEF	United Nations Children’s Fund
USAID	United States Agency for International Development
VIF	Variance Inflation Factor
WASH	Water, Sanitation and Hygiene
WHO	World Health Organization
WHZ	Weight-for-Height Z-score
WPRO	Western Pacific Region
